# The continuing role of communities affected by HIV in sustained engagement in health and rights

**DOI:** 10.1002/jia2.25724

**Published:** 2021-06-30

**Authors:** Georgina Caswell, Vuyiseka Dubula, Solange Baptiste, Helen Etya’ale, Omar Syarif, David Barr

**Affiliations:** ^1^ Global Network of People Living with HIV Cape Town South Africa; ^2^ Centre for Civil Society University of KwaZulu‐Natal Durban South Africa; ^3^ International Treatment Preparedness Coalition Johannesburg South Africa; ^4^ Global Network of People Living with HIV Amsterdam The Netherlands; ^5^ The Fremont Center New York United States of America

**Keywords:** people living with HIV, community engagement, research, service delivery, accountability, universal health coverage

## Abstract

**Introduction:**

The meaningful involvement of persons affected by a disease is a unique aspect of the HIV response that places people living with (PLHIV) and those directly affected by HIV (peers) at the centre of the design, development and implementation of service delivery and research and policy making. The principle of greater involvement of PLHIV (GIPA) has and will increasingly ensure equitable access to services and engagement of marginalized groups in the HIV response, and to health services more broadly. This paper describes the history, current place in the HIV response and potential future role of PLHIV and communities in health responses.

**Discussion:**

Historically, the role of communities of PLHIV and peers in service delivery, research and drug development, advocacy, social and political accountability, resource mobilization and social and human rights protection is well documented. Their leadership and engagement have contributed directly to improved outcomes in access to HIV treatment, prevention, support and care services around the world. Their continued and expanded role is especially important for the future success of HIV responses in sub‐Saharan Africa, where the HIV burden remains the greatest. The lessons learned from the leadership and involvement of communities of PLHIV and peers in the HIV response hold value beyond HIV responses. The models and approaches they have efficiently and effectively utilized have relevant applications in addressing shortfalls in health systems in the COVID‐19 era, as well as broader, more integrated health challenges as countries move to develop and operationalize universal health coverage (UHC). However, neither HIV nor other health and development targets can be met if their contributions are not adequately recognized, valued and funded.

**Conclusions:**

The past three decades have demonstrated that communities of PLHIV and their peers are instrumental in sustaining engagement and advocacy for health equity and financing for health and ensuring that the human rights of all people are recognized and upheld. Quality and effective integration of health systems and UHC can be more effectively designed, implemented and sustained with communities of PLHIV and peers at the centre.

## 1 INTRODUCTION

The principle of meaningful and greater involvement of people living with HIV/AIDS (GIPA) was first articulated at a conference in the United States [1] and then expanded globally [2]. It has been the cornerstone of efforts by activists at global, regional and national levels to ensure more effective, higher quality HIV programming and services that meet the needs of all people living with HIV (PLHIV) – including the most marginalized – in rights‐based, equitable, destigmatizing ways [3]. In practice, the application of GIPA is commonly seen in the coordination, leadership and work of networks of PLHIV, community‐based organizations, key population networks and other civil society bodies. These groups play a crucial role in the HIV response throughout sub‐Saharan Africa and elsewhere. However, the impact is often limited due to lack of funding and legal, policy and political barriers.

We argue in this commentary that increased and sustained leadership and engagement of communities across HIV prevention, treatment and care services is essential for future progress in fighting the epidemic. We define “community” as groups of people with shared interests, behavioural norms and/or common geographical location [4] – and, within a health context, to people, organizations and networks that enable rights‐based access to services for recipients of care. For this paper, the community of focus is PLHIV and people within the overall definition who are at high risk of or directly affected by HIV.

This paper describes the history of community involvement, the current contributions in the HIV response and the potential future role of communities of PLHIV in health, including in helping ensure universal health coverage (UHC) that is equitable and responsive to the needs of PLHIV and those most affected. In UHC and other priorities for integration of health systems, communities of PLHIV offer lessons for community‐led, person‐centred health and participation in social protection that go beyond a single‐disease focus.

## 2 DISCUSSION

### 2.1 The early days: defining an identity of “living with AIDS”

The creation of an identity of people “living with AIDS” was crucial at a time when AIDS was considered to be a death sentence. Openly declaring this identity gave people living with AIDS hope; it demanded that they be treated with dignity and respect, and it gave them control. The image of the empowered people living with AIDS was an important source of strength for both the individual and the wider community of people living with AIDS – in sharp contrast to media images portraying people living with AIDS as helpless victims.

Prominent examples of this “community by identity” were forged around groups driven by PLHIV and people most at risk, such as The AIDS Support Organization (TASO) in Uganda and the AIDS Coalition To Unleash Power (ACT‐UP) in the United States, both founded in 1987. These groups focused on providing support services for PLHIV, prevention education and advocacy to propel governmental responses to the epidemic.

The South Africa‐based Treatment Action Campaign (TAC), formed in December 1998, is one of Africa’s best‐known activist groups. TAC initially focused on forcing society to recognize and acknowledge PLHIV. At a time when HIV stigma was highest in South Africa and disclosing one’s HIV status was potentially deadly, TAC’s use of a T‐shirt emblazoned with a bright “HIV positive” exemplified personal and community empowerment. A simple T‐shirt slogan connected PLHIV to a community, leading them to education, support, advocacy, influence and power.

As a community, PLHIV and those affected by HIV in South Africa, elsewhere in Africa and globally were not only saying that their lives were worth saving and that more action must be taken on their behalf, but also demanding seats at the table in HIV programming and policy making. Direct and meaningful engagement became a central organizing principle for the community’s response to HIV on micro and macro levels, as it remains today.

As illustrated in examples herein and beyond, the work and impact of communities have been demonstrated through lobbying for greater access to quality antiretroviral therapy (ART) [5], delivering services outside facilities [6, 7], financing community organizations [6], influencing national and regional policy [6], driving evidence‐based accountability initiatives [6, 8], addressing individual and social level HIV‐related stigma [9] and promoting treatment access as a human right [10]. Communities’ advocacy for, design of and implementation of “safer sex” and harm reduction principles and approaches from the beginning of the epidemic have prevented countless new HIV infections. Treatment adherence support and treatment literacy programmes – many first created, expanded and then differentiated for local conditions and community needs – have helped initiate and retain PLHIV on ART by providing critical assistance and information. This has enabled them and their families to understand how and why ART works and how they can best manage side‐effects, access difficulties, confidentiality and stigma [11].

Advocacy for affordable drug pricing has contributed to countries and donors dramatically scaling up access to ART and other essential medicines [12]. Advancements in affordability of drugs and other key commodities contribute directly to other outcomes, including fewer new infections and increased testing uptake, as the availability of treatment often incentivizes people to test for HIV.

### 2.2 Common current roles of communities in the HIV response

Communities continue to champion the needs and rights of PLHIV through other service delivery and broader accountability workstreams, as detailed below and in Table 1.

**Table 1:** Illustrative examples of the work and reach of communities of people living with HIV (PLHIV) and peers

**Service delivery**	
*The AIDS Support Organisation (TASO)* *Uganda*	In 2018, TASO provided HIV counselling and testing for more than 600,000 people, care and support to more than 65,000 orphans and vulnerable children and antiretroviral therapy (ART) to more than 200,000 people [26].
*Treatment Action Campaign (TAC)* *South Africa*	TAC activists and their allies’ continued advocacy, vigilance and contestation of HIV policies led to the largest ART programme in the world, with over five million people on treatment by 2019. Its support for treatment literacy and adherence has helped sustain a relatively high viral suppression level among all those on ART (at 92% in 2019) [27].
*READY+ (Frontline AIDS, Y+ Global, AFRICAID/Zvandiri, CANGO, GNP+, M&C Saatchi World Services, PATA and REPSSI)* *Mozambique, Eswatini, Tanzania and Zimbabwe*	Since 2016, READY+ has been reaching adolescents and young people living with HIV to increase access to holistic care and support. It promotes not only sexual and reproductive health and rights (SRHR), but also mental health in order to foster resilience. Community adolescent treatment supporters (CATS) play a vital role. During home and clinic visits, CATS provide information, counselling and support to other young people living with HIV and encourage adherence to HIV treatment [28].
*Kenya Legal & Ethical Issues Network on HIV and AIDS (KELIN)* *Kenya*	In 2019, KELIN opposed intellectual property laws that impede access to tuberculosis and HIV drugs. The organization also focused on creating an enabling environment for the realization of SRHR in Kenya for women and girls, who are disproportionately at risk for HIV in the country; this included training young women on their SRHR rights [29].
**Community‐led research**	
*ITPC Middle East and North Africa (ITPC MENA)* *Egypt, Morocco, Tunisia*	In 2017, ITPC MENA conducted a research study on the impact of provisions in intellectual property legislation on access to medicines in Egypt, Morocco and Tunisia. Provision categories included ability to extend patents, patent opposition, use of compulsory licences and parallel imports of medicines. The intellectual property landscape was assessed to be least restrictive for access to medicines in Egypt and most restrictive in Morocco. The study provides a framework for a similar analysis in other countries [30].
*National Network of Positive Women Ethiopians (NNPWE)* *Ethiopia*	NNPWE investigated access to reproductive health services among women living with and at risk of HIV. The research findings across several major cities in Ethiopia revealed access gaps for the prevention of mother‐to‐child transmission (PMTCT) services. NNPWE’s recommendations and subsequent advocacy have focused on better integration between antenatal and PMTCT services, improvement of poor service conditions and implementation of WHO treatment guidelines [31].
*Global Network of Sex Worker Projects (NSWP)* *Global*	NSWP led a global research initiative that included, among others, in‐country research (ten countries) and a global e‐consultation (ten countries). It investigated the impact of stock‐outs of essential medicines (including for ART), condoms and lubricants and diagnostic testing supplies for STIs, HIV and viral load levels. The potential consequences highlighted included treatment interruptions, increased vulnerability to infections, HIV/STI‐related resistance and treatment failure [32].
**Advocacy**	
*Southern African AIDS Trust (SAT), Zimbabwe AIDS Network (ZAN) and AIDS Accountability International (AAI)* *Zimbabwe*	In 2013, in an effort to achieve greater and more meaningful engagement of civil society in the Global Fund to fight AIDS, Tuberculosis and Malaria (Global Fund) concept note development process in Zimbabwe, SAT, ZAN and AAI coordinated a civil society coalition. In a workshop that brought together a diverse group of key populations, PLHIV, young women and people with disabilities, participants agreed on a priority charter – a landmark consensus document on priorities for the national response to tuberculosis. The tool was used as an advocacy tool to hold the government and the Global Fund country coordinating mechanism accountable during the process of developing the concept note [6].
*Positive Vibes* *Namibia*	In 2012, Namibia attained upper‐middle‐income status, which came with the threat of the phasing out of international funding from donors, including the Global Fund and the US President’s Emergency Plan for AIDS Relief (PEPFAR). Using the strategic investment approach, a group of civil society organizations demonstrated the comparative advantage of how communities can be involved in the design and delivery of an effective HIV response. Positive Vibes led the drafting of a position paper that established a platform for dialogue with the government and coordinated investment efforts around community HIV responses [6].
**Community‐led monitoring**	
*Malawi Network of Religious Leaders living with AIDS (MANERELA+), Community Initiative for Tuberculosis, HIV/AIDS and Malaria plus related diseases (CITAM+), Zimbabwe National Network of People Living with HIV (ZNNP+)* *Malawi, Zambia, Zimbabwe*	These three national PLHIV networks in Malawi, Zambia and Zimbabwe implemented community treatment observatories (CTOs), collecting data systematically on the uptake of differentiated service delivery (DSD) models and access to routine viral load testing for recipients of care. Implementation of the CTO in Zambia led to the inclusion of CITAM+ in the national DSD technical working group. Meanwhile, the value of CTOs was recognized in Malawi and Zimbabwe, with the model integrated in Zimbabwe’s 2020–2025 National Strategic Plan and Malawi’s PEPFAR Country Operational Plan [33].
*Positive Generation* *Cameroon* *Reseau Accès Aux Médicaments Essentiels (RAME)* *Burkina Faso*	These community health observatories in Cameroon and Burkina Faso use selected recipients of care and community health workers, known as *sentinelles*, who support communities in accessing health services and report any grievances or health facility‐level dysfunctions to organizers. The operations of *sentinelles* highlighted and publicized incidents of HIV‐related stigma at health facilities in Cameroon and instances where recipients of care were being wrongfully asked to pay fees for HIV services in Burkina Faso [34].
*Global Network of Young People Living with HIV (Y+)* *Global*	Using a scorecard, young people living with HIV are documenting their experiences of using and accessing health services, and the data are being shared back with health facilities in a facilitated conversation. This has led to the development of quality improvement plans in Mozambique and Zimbabwe that are being used to improve health services [35, 36].

#### 2.2.1 Service delivery

Communities of PLHIV work synergistically with health systems to bring into and retain in care marginalized populations, which are often difficult to reach without the specific experience and expertise that only community‐led services can provide. At a basic level, it is as simple as a person living with HIV, fearful of being stigmatized or scared of being ill, feeling more comfortable with someone who has “been there.” For these reasons and others, a World Bank study of HIV service delivery concluded that community‐based efforts are a “foundation” of the response to HIV/AIDS and generated significant outcomes in return for the financial investment in the sector [13].

Not only do communities generate demand for services, but they also design, lead and support HIV provision. Specifically, they carry out HIV pre‐ and post‐test counselling, home‐based care, delivery of medicines, health education, adherence support through specialized peer‐based adherence clubs and sending SMS reminders. Often, and crucially, their work is also closely tied with clients’ overall health and wellbeing beyond biomedical responses to HIV, including referrals for tuberculosis screening, treatment for sexually transmitted infections (STIs), mental healthcare support and legal services [14].

Evidence shows that peer‐led service delivery enhances the reach, uptake and quality of HIV services, as well as the dignity, quality of life and retention in care of PLHIV. The presence of peer workers in clinics has been reported to reduce waiting times, streamline recipient of care flow and lighten excessive workloads among facility staff [15]. In Senegal, peer mentoring for HIV counselling and testing was found to cause an increase in testing of HIV‐positive partners. In South Africa, a randomized controlled trial found that people living with HIV with peer adherence support were more consistent in picking up their medication, attaining a treatment pick‐up rate of 95% compared with 67% among those without such support [16].

Recent examples from activities initiated or altered during the COVID‐19 pandemic also demonstrate community adaptability, flexibility and innovation in ensuring that PLHIV are retained in treatment and care. Members of the Uganda Network of Young People living with HIV/AIDS, for example, continued to deliver medicines on bicycles to minimize treatment interruptions. In Zimbabwe, Africaid‐Zvandiri’s community adolescent treatment supporters provided online counselling and support to their peers on coping with the impact of the virus [17].

#### 2.2.2 Community‐led accountability activities

When properly resourced, communities can undertake and deliver crucial research and analytical work aimed at ensuring that health systems, governments and other stakeholders are accountable for meeting all the needs of PLHIV, including upholding human rights. They do this through community‐led research, monitoring and advocacy activities at local, regional and national levels [6, 8] (Table 1).

When communities participate directly in research, including as partners with traditional academic researchers, their engagement as “co‐creators” and subjects of research can play a critical role in improving the quality of research design and analysis. Communities can ensure that research is grounded in reality and lived experiences, sensitive to and supportive of participants involved and that the analysis is context‐specific [18]. But communities also devise and undertake high‐value research of their own, further identifying the specific health and human rights priorities and challenges that must be addressed for improved HIV responses [19].

Community‐led research helps inform policy makers and programme planners about:

1. The experiences and needs of the communities that policies and programmes aim to reach.
2. The accessibility, affordability, quality and effectiveness of the services and policies they are delivering.

The Stigma Index 2.0 of the Global Network of People Living with HIV (GNP+), for example, is a key tool for understanding the persistent effects of HIV‐related stigma in different settings (e.g. household and workplace) and identifying gaps that must be addressed in policies and programmes to ensure stigma‐free access to health and support services [20]. The research tool was conceptualized, designed and implemented entirely by communities. The results from the PLHIV Stigma Index research have been used to hold governments accountable for upholding HIV and human rights commitments, such as those in the 2016 United Nations Political Declaration on Ending AIDS.

Community‐led advocacy and campaigning have mobilized millions of individuals around various objectives, including: influencing policies and laws; improving access to prevention, treatment, care and support; reducing stigma and discrimination; exposing corruption; and creating more enabling environments for PLHIV and others affected by HIV. The Undetectable=Untransmittable (U = U) campaign, launched in 2016 to help reduce stigma and misinformation, is an example of a coordinated, global, community‐developed and community‐driven advocacy effort that has mobilized and involved more than 400 community partners in over 60 countries [21]. In sub‐Saharan Africa, knowledge of U = U has grown and is poised to grow further with tailored, context‐specific packaging [22, 23].

At local levels, advocacy includes legal services, ombudsmen services within health facilities, training for law enforcement agencies and lobbying for human rights protections. TAC again provides a significant example of the power of PLHIV advocacy through its combination of street demonstration and legal action leading to the development and scale‐up of South Africa’s national ART programme [24].

PLHIV are also now incorporated into public health policy and resource mobilization efforts at national and global levels, including participation on the board and country coordinating mechanisms of The Global Fund to Fight AIDS, Tuberculosis and Malaria and participation in the drafting of annual US President’s Emergency Plan for AIDS Relief (PEPFAR) country operational plans. PLHIV are also members of data and safety monitoring boards and community advisory boards for clinical research programmes, the UNAIDS Programme Coordinating Board and the UNITAID Board, and participate in processes to develop World Health Organization (WHO) guidelines.

The involvement of PLHIV has also transformed clinical research and drug development. The late 1980s saw PLHIV activists lead a successful movement to compel the US Food and Drug Administration (FDA) to shorten its drug‐approval process, facilitating entry into the market of new drugs to treat HIV infection and, by the mid‐1990s, of successful ART regimens [5]. Once again, PLHIV activists are driving the call for equitable distribution and access to COVID‐19 vaccines in Africa and other resource‐limited settings [25].

The implementation of various community‐led monitoring mechanisms (e.g. health facility committees, citizen report cards, community report cards and community observatories) led to site‐ and systemic‐level changes and improved healthcare access for PLHIV, as well as key and vulnerable populations. Through the Regional Community Treatment Observatory (RCTO) developed and supported by the International Treatment Preparedness Coalition (ITPC), PLHIV networks systematically collected quantitative and qualitative data across the HIV cascade through a specially designed model that included 103 indicators in areas such as viral load testing uptake, disaggregated by various factors (including age, sex and key population association) to achieve useful granularity. Coupled with advocacy, the RCTO led to, among others, the revision of the central pharmacy dispatching system in Senegal, adoption of a new differentiated service delivery policy in Sierra Leone and elimination of user fees for health services in Côte d’Ivoire [8].

#### 2.2.3 PLHIV communities in current and future integrated systems for health

In 2018, the ITPC‐led Make Medicines Affordable consortium catalysed an average price reduction of 67% across 15 target antiretroviral drugs in four middle‐income countries, contributing to a total annualized financial benefit of over $300 million [37]. Such advocacy has significantly impacted the affordability of diagnostics and medicines, reaching beyond HIV – and now carried over to medicines for cancer, diabetes, tuberculosis, hepatitis and, most recently, COVID‐19 treatment and vaccine costs.

Such linkages and influence outside of specific HIV responses underscore the value of the models developed by PLHIV communities as service providers, advocates and monitors in integrated health systems. There are both opportunities and potential challenges. More integrated approaches will allow advocates to address disparities in access to healthcare that cannot be improved within the confines of an HIV‐specific lens. Integration also provides more opportunities for health systems to better incorporate the lessons learned from PLHIV communities into care beyond the HIV silo.

But the dangers are also great. Ensuring that such transitions to more integrated systems do not undermine the progress made in HIV to date, weaken human rights protections or increase rather than reduce inequities will require intensive advocacy and monitoring from PLHIV communities [38, 39]. This is why GNP+ and other global key population networks have been advocating for policy makers to “put the last mile first”. As GNP+ says: “The logic, and moral obligation, is clear. If universal health coverage works for the poorest and most marginalized – including people living with HIV and other key and vulnerable communities (who are directly and disproportionately affected by diseases and poor health) – it will work for everyone.” [40]

Realizing the potential of communities to do this work requires them to be better resourced, with increased and more consistent financing available [41]. Although one of the UNAIDS fast‐track commitments towards ending AIDS by 2030 is “ensuring that at least 30% of service delivery is community‐led” [42], little progress has been made towards achieving this commitment in any part of the world. A deliberate effort is needed to track and increase funding for community‐led efforts as a component of health systems.

But money alone is not enough. To fully realize the value of communities in achieving health outcomes, the public health establishment needs to value this work on par with health, academic and government systems. Ultimately, it is the end‐user that determines the success of any public health intervention.

## 3 CONCLUSIONS

If individual and community engagement in health is key to good public health outcomes, then the HIV experience is the greatest example of such engagement. As the global public health infrastructure turns towards more integrated models to meet UHC goals and as the world copes with a new and deadly pandemic, the shape of the HIV epidemic in sub‐Saharan Africa will shift to an epidemic that primarily affects key populations – with the work of PLHIV, peers and allies offering important lessons about how to treat people with dignity, improve health outcomes and defend human rights.

## Competing interests

The authors have declared no conflict of interest.

## Authors’ contributions

GC, VD, SB, HE, OS and DB contributed to conceptualizing and writing the paper.

## Funding

This article is part of a supplement and any costs related to the publication of this article are covered by the supplement organizers.
